# Prophylactic Interventions for Heel Pressure Ulcers in Critically Ill Patients Admitted to an Intensive Care Unit: A Systematic Review and Meta-Analysis

**DOI:** 10.7759/cureus.83029

**Published:** 2025-04-26

**Authors:** Mayumi Koyanagi, Hideaki Sakuramoto, Kohei Kajiwara, Ayako Fukushima, Shun Yoshihara, Megumi Mukoyama, Megumi Horinouchi, Aiko Mihara, Yuta Imamura

**Affiliations:** 1 Department of Nursing, Fukuoka Tokushukai Hospital, Kasuga, JPN; 2 Division of Faculty Development and Nursing, Kindai University, Osaka, JPN; 3 Faculty of Nursing, Shimonoseki City University, Shimonoseki, JPN; 4 Department of Critical Care and Disaster Nursing, Hokkaido University of Science, Sapporo, JPN; 5 Faculty of Nursing, Japanese Red Cross Kyushu International College of Nursing, Munakata, JPN; 6 Department of Nursing, Japanese Red Cross Fukuoka Hospital, Fukuoka, JPN; 7 Department of Nursing, University of Occupational and Environmental Health, Kitakyushu, JPN; 8 Department of Nursing, Kurume University Hospital, Kurume, JPN; 9 Department of Nursing, National Hospital Organization Kumamoto Medical Center, Kumamoto, JPN

**Keywords:** critical care, heels, icu, pressure ulcers, preventive intervention

## Abstract

Pressure ulcers are one of the most predictable adverse events, and nurses play a major role in their prevention. Patients receiving intensive care are at a particularly high risk of developing pressure ulcers. The heel is a common area for pressure ulcers, but the number of comparative studies on preventing pressure ulcers is small. Therefore, it is important to combine the evidence. This study aimed to investigate the effectiveness of interventions to prevent heel pressure ulcers by comprehensively extracting and quantitatively integrating the results of studies on preventive care for heel pressure ulcers in critically ill patients.

Systematic reviews and meta-analyses were performed. This systematic review was conducted in accordance with the Preferred Reporting Items for Systematic Reviews and Meta-Analyses guidelines (PRISMA) 2020. Eligible criteria were randomized controlled trials (RCTs) and non-RCTs examining interventions for the prevention of heel pressure ulcers as an adjunct to standard care in critically ill adult patients in intensive care units. According to the eligibility and exclusion criteria, two review authors independently screened and extracted data from the included literature. The risk of bias was assessed using the Mixed Methods Appraisal Tool (MMAT). Studies were grouped based on intervention type, and a random-effects meta-analysis was performed.

A total of 2,800 studies were searched in four databases. In the primary screening, 122 met the eligibility criteria, and in the secondary screening, seven studies (1,412 patients) met the eligibility criteria. Of the seven studies, three (1,037 eligible patients) applied dressings to the heel, two (237 eligible patients) applied oil, and two (138 eligible patients) wore heel protectors. The risk of bias of the included studies was low. Preventive intervention for heel pressure ulcers significantly reduced the incidence of pressure ulcers compared with usual care (odds ratio (OR) = 0.16, 95% confidence interval (CI) = 0.08-0.33, I² = 0%)

In the subgroup analysis, dressing was effective in preventing heel pressure ulcers (three studies, OR = 0.15, 95% CI = 0.05-0.45, I² = 0%). Protector was effective in preventing heel pressure ulcers (two studies, OR = 0.15, 95% CI = 0.05-0.45, I² = 0%). However, oil application was not significantly effective in preventing pressure ulcers (two studies, OR = 0.34, 95% CI = 0.06-1.90, I² = 0%). In this subgroup analysis, oil application alone was ineffective in preventing pressure ulcers. This number of studies is insufficient to draw firm conclusions, and further studies are required.

## Introduction and background

According to the international guidelines, pressure ulcers are defined as localized damage to the skin and/or underlying tissue resulting from pressure, or pressure in combination with shear [1]. That is, it is the result of tissue damage due to inadequate tissue perfusion and is considered one of the preventable adverse events. One in 10 adult patients admitted to the hospital is affected by pressure ulcers, suggesting that the burden of pressure ulcers is large [2].

Patients in intensive care, in particular, are often forced to rest due to their reduced level of consciousness, as well as unstable circulatory dynamics and respiratory status caused by shock and severe circulatory failure. In addition, medical equipment such as ventilators, percutaneous cardiopulmonary support (PCPS), and aortic balloon pumping is often inserted, which restricts body movement. They may also be immobile due to the use of sedatives, often undernourished due to fasting or inability to eat, and have significant edema - making them especially vulnerable to pressure ulcers. Therefore, preventive care is essential [3].

In an international survey of intensive care units (ICUs) conducted in 1,117 facilities across 90 countries, the prevalence of pressure ulcers in ICUs was reported to be 16.2% (95% confidence interval (CI): 15.6%-16.8%) [4]. In addition, the prevalence of pressure ulcers by site was 26.9%-48.0% for the sacrum, 18.5%-38.9% for the heel, 4.1%-46.4% for the buttocks, 10.9%-15.7% for the hip joint, 4.3%-19.7% for the ear, and 0.0%-40.2% for the shoulder [5]. The prevalence of pressure ulcers by site is said to be the second highest in the heel, after the sacrum.

The lower extremities are the furthest from the heart and are susceptible to circulatory failure, such as shock and low blood pressure. The heel has a unique structure, with little subcutaneous tissue for protection and no muscles or fascia, making it vulnerable to pressure, friction, and shear forces. In addition, ICU patients often experience decreased perception and mobility due to sedation and paralysis, are prone to edema, and are frequently subjected to prolonged pressure in a lying position - making them more susceptible to necrosis due to impaired blood flow and embolism. Based on these findings, the heel is an important site for pressure ulcer prevention, and there may be prevention methods specific to ICUs [6].

In a recent meta-analysis [7] conducted in 2021 regarding the prevention of pressure ulcers in ICUs, the effect of applying a prophylactic dressing on the heel showed a risk ratio of 0.31 (95% CI: 0.12-0.80), based on the results of randomized controlled trials (RCTs) up to 2019. In addition, as a direct method to prevent pressure ulcers on the heel, there are approaches such as applying oil to reduce friction and using heel protectors for preventive care. Previous studies have reported the use of oil as a preventive treatment for pressure ulcers in the heel area, but the subjects were not severely ill, and there were no reports of meta-analyses. Meta-analyses of pressure ulcer prevention interventions, including heel protectors, have been conducted for acute care beds [8], but not for ICU patients. However, in 2017, an RCT [9] on pressure ulcer prevention interventions using heel protectors was conducted, but these RCTs were not included in previous meta-analyses. In summary, while intervention studies on the prevention of heel pressure ulcers using dressings have been conducted - and even a meta-analysis exists - the number of systematic reviews remains insufficient, and the evidence base is not well-established. Furthermore, the efficacy of interventions targeting the heel - such as dressings, oil applications, and protectors - remains unclear, despite the high risk of pressure ulcers in ICU patients. To prevent heel pressure ulcers in critically ill patients, improving the quality of care requires a comprehensive review and analysis of prior studies to identify effective interventions.

The aim of the study was to investigate the effectiveness of interventions to prevent heel pressure ulcers by comprehensively extracting the results of research on preventive care for heel pressure ulcers in critically ill patients, and quantitatively integrating them.

## Review

Materials and methods

Study Design and Protocol Registration

This was a systematic review and meta-analysis. It was conducted in accordance with the Preferred Reporting Items for Systematic Reviews and Meta-Analyses (PRISMA) Statement (Appendix Table 3) [10]. The study was registered with the Open Science Framework (https://osf.io/r87gc/).

Research Questions

The research questions were set in line with the Population, Intervention, Comparison, and Outcome (PICO) framework. The population was defined as adults aged ≥18 years who were critically ill in all departments, including surgery, internal medicine, and emergency departments. The interventions included receiving direct preventive care for the heel, such as wearing a heel protector, in addition to routine care. The control group included patients who received routine care, such as regular body repositioning, pressure ulcer risk assessment, nutritional support, and bed mattress selection. The incidence of calcaneal pressure ulcers was the primary outcome of this meta-analysis.

Search Strategy

The databases extracted articles from PubMed, the Cochrane Central Register of Controlled Trials, the Cumulative Index to Nursing and Allied Health Literature (CINAHL), and the Central Journal of Medicine, with a limited focus on Japanese and English papers published up to July 2024. Controlled vocabulary was created by converting terms into appropriate equivalents for each database, based on basic conditions (study location, study design, and intervention method). The following search terms were used: intensive care unit, pressure ulcer, prevention, randomized, and non-RCT. Further details are provided in Appendix Table 4.

Eligibility Criteria

In this study, differences and details related to the study area, environment, and variations in beds and mattresses were not included in the endpoints. We included literature that measured the incidence of new pressure ulcers by providing direct intervention to the heel in addition to routine care, compared with the provision of routine care alone. The eligibility criteria were as follows, in accordance with the PICO framework set as a basic condition: (1) the target population is adults hospitalized in the ICU and critically ill, regardless of condition, disease, or prognosis; (2) the intervention is direct preventive care for heel pressure ulcers; (3) the research setting is the ICU; (4) the study design may be an RCT, non-RCT, before-after comparative trial, or intervention study. The exclusion criteria were as follows: (1) studies written in languages other than Japanese or English; (2) literature without a control group.

Outcomes

Our primary outcome was the incidence of heel pressure ulcers in hospitals. As an intervention, we included literature from intervention studies that provided pressure ulcer prevention care directly to the heel in addition to usual care, which was considered an important outcome for critically ill patients in this study. In addition to the inclusion and exclusion criteria, we extracted data from the literature based on the criteria for pressure ulcers. In this study, the severity of pressure ulcers was not assessed, and outcomes were judged solely by whether pressure ulcers occurred.

Study Screening and Data Extraction

The article identified by the search was imported into Rayyan.ai software (Qatar Computing Research Institute, Ar-Rayyan, Qatar; http://rayyan.qcri.org/). As a primary screening, two reviewers screened the article individually to confirm that the basic conditions were met, based on the title and abstract of the article, and the evaluations of the two researchers were collated. In the event of a conflict between the two evaluations, a third reviewer decided whether to accept the proposal. In addition, if a clear judgment could not be made, the article was adopted. In the secondary screening, the full text of the article was obtained, and two reviewers screened the study individually to ensure that the study design, target audience, and intervention met the basic criteria. If the eligibility criteria were met, the study was accepted. In the event of a conflict between the two evaluations, a third reviewer decided whether or not to accept the proposal.

Articles accepted by secondary screening were independently extracted by two review authors based on the following: year of publication, country, ICU type, participant age, duration of ventilator use, ICU admission time, intervention method, and the presence or absence of heel pressure ulcer formation.

Assessment of the Risk of Bias

The studies included not only RCTs but also non-RCTs. Therefore, the evaluation was conducted using the Mixed Methods Appraisal Tool (MMAT), version 2018 [11,12]. There were two common items: (1) Do you have a clear research topic? and (2) Can the collected data address this research problem? RCTs and non-RCTs were evaluated on these two common items using three levels: “Yes,” “No,” and “Can’t tell.” If “No” or “Can’t tell” was selected for either of the two common items, the article was excluded as not being an empirical study. In addition, if both common items were evaluated as “Yes,” the five criteria of each study design were evaluated on the same three terms: “Yes,” “No,” and “Can’t tell.” The five items for RCTs were as follows: (2.1) whether the study participants were adequately randomized, (2.2) whether the intervention and control groups were comparable at the start of the study, (2.3) whether complete outcome data were available, (2.4) whether outcome assessors were blinded to the intervention, and (2.5) whether participants were able to comply with the assigned intervention. The five items for non-RCTs were as follows: (3.1) participants were representative of the target population, (3.2) measured variables were measured in a clear and credible manner, (3.3) complete outcome data were available, (3.4) confounders were taken into account in the design and analysis, and (3.5) whether the intervention was implemented as intended during the study period. The quality of the studies was then critically assessed based on the number of “No” and “Can’t tell” responses to the five items in each study design. We also examined publication bias using funnel plots. However, when the number of articles included in the study was small (fewer than 10), the shape of the plots tended to be unstable. It is also difficult to accurately determine the presence or absence of asymmetry or bias, making visual interpretation challenging. Furthermore, the results tend to depend on specific conditions and contexts, making it difficult to draw broad conclusions and limiting the generalizability of the results [13,14]. Therefore, we decided not to evaluate the risk of bias, as we believe that the potential error in assessing the risk of bias would be greater.

Data Synthesis and Assessment of Heterogeneity

The synthesis and analysis of the data extracted from the article use the method of meta-analysis, and the analysis software, which is the graphical user interface of R (The R Foundation for Statistical Computing, Vienna, Austria), is EZR (Saitama Medical Center, Jichi Medical University, Saitama, Japan). EZR is a graphical user interface for R, or more precisely, an improved version of the R Commander, designed to add statistical functions frequently used in biostatistics.

Two variables that are conceptually homogeneous are defined as odds ratios (ORs). Seven articles were adapted for this systematic review, but the details of the methodological differences between the articles were not included in the evaluation. Random Effects Meta-analysis was chosen to allow for differences in research methodology and approach across articles. The study also aimed to identify the effectiveness of interventions to prevent heel pressure ulcers by using statistical methods to investigate the effectiveness of such interventions, comprehensively identifying and quantitatively integrating studies on preventive care for heel pressure ulcers in critically ill patients. ORs were also evaluated to compare the likelihood of developing pressure ulcers between the intervention group, which received the pressure ulcer prevention intervention, and the control group, which did not. A 95% CI was calculated for the reliability of the statistics. The effect size was evaluated based on previous studies [15]. Data were analyzed in subgroups by intervention methods and study design and pooled by forest plots. Variation in conclusions across articles was determined using the heterogeneity I² statistic. According to the Cochrane Handbook [16], “heterogeneity may not be significant” in the case of I² < 40% of cases. Therefore, I² ≧ 40% was judged to be highly heterogeneous. We also conducted a sensitivity analysis of each type of intervention to assess the robustness of the data.

Results

An article search was conducted on July 28, 2024. A total of 514 studies from PubMed, 2,137 from the Cochrane Central Register of Controlled Trials, 415 from CINAHL, and 204 from the Central Journal of Medicine were identified, for a total of 3,270 studies. Of these, 2,800 articles, excluding duplicate studies, were screened.

**Figure 1:**
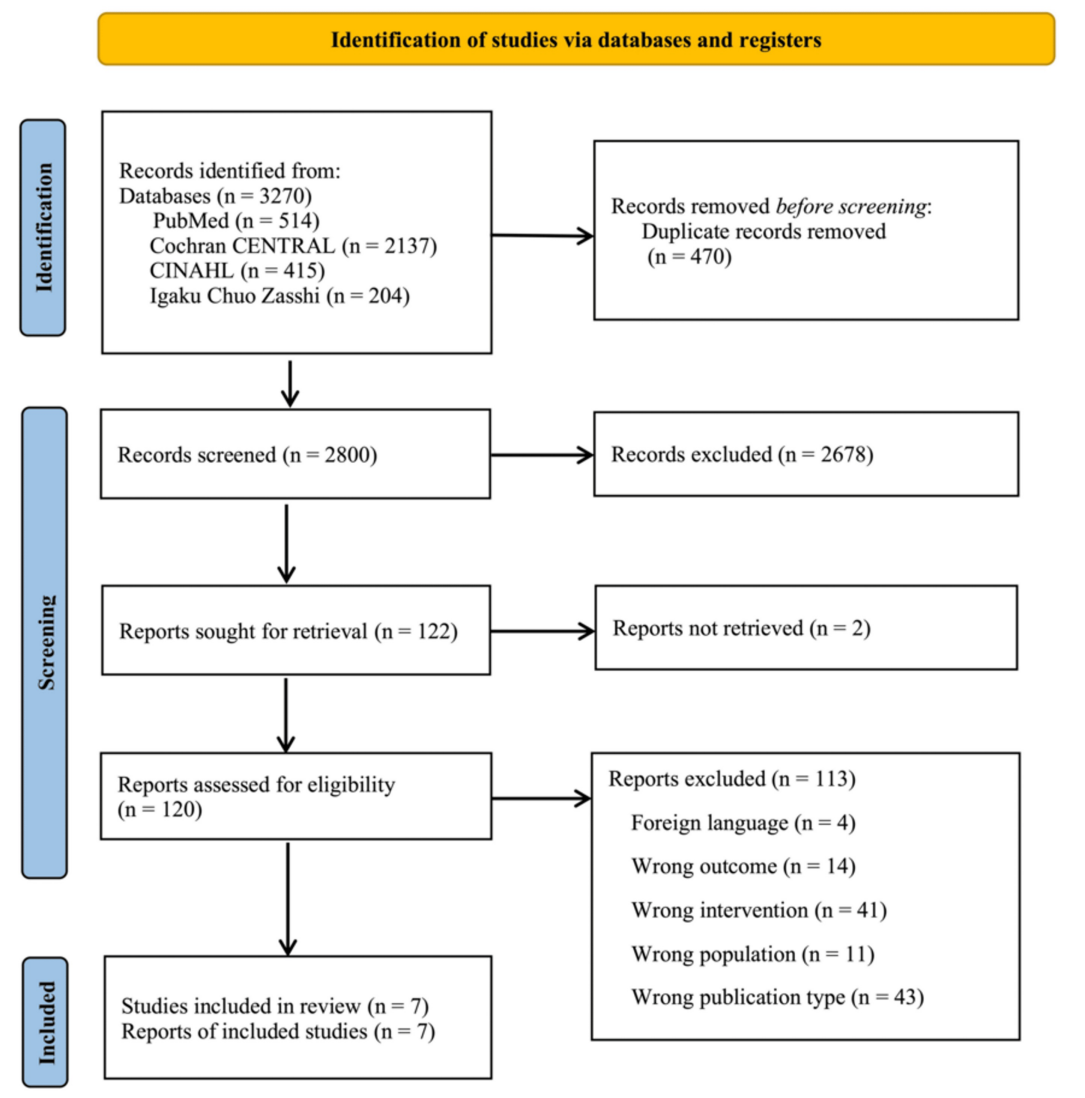
Preferred Reporting Items for Systematic Reviews and Meta-Analyses flow diagram The selection of studies on prophylactic interventions for heel pressure ulcers in critically ill patients, admitted to the intensive care unit, is presented. Abbreviation: Cochrane CENTRAL, Cochrane Central Register of Controlled Trials; CINAHL, Cumulative Index to Nursing and Allied Health Literature

In the first screening, 2,678 cases were excluded. In the second screening, 122 full-text studies were searched, and eligibility was assessed according to the inclusion and exclusion criteria. Seven of these studies met the eligibility criteria (Figure 1).

Characteristics of the Study

The characteristics of the seven selected studies are summarized in Table 1. The included studies were published between 2013 and 2024 and were conducted in Turkey (two studies), Iran (one study), Germany (one study), the United States (two studies), and Australia (two studies). Six of the seven studies were RCTs, and one was a case-control study. A total of 1,412 patients (739 in the intervention group and 673 in the control group) were included in the study, with a mean age of 40.6-74.52 years. The subjects included three studies on trauma emergency patients and four studies in the general intensive care field, including surgery, internal medicine, and cardiology. Four of the seven studies involved patients on mechanical ventilation or sedation for at least 90%, and five of the seven studies had ICU stays of ≥3-5 days. As an inclusion criterion, four studies used the Braden scale at the time of entry, with one criterion being a Braden scale score of ≤18. There was also one study that looked at sedatives and immobility time as criteria. As exclusion criteria, some literature excluded patients with peripheral vascular disease, unstable peripheral circulatory dynamics, patients receiving vasoconstrictor drugs, and patients who were contraindicated for repositioning for medical reasons. In addition, two studies in trauma emergency patients excluded patients with spinal cord injuries or those with a history of pressure ulcers or trauma in the heel.

**Table 1:** Characteristics of the included studies This table details the characteristics, extracted data, and interventions of seven studies that met the inclusion criteria. Standard care includes risk assessment, regular repositioning, nutritional support, incontinence management, selection of bed mattresses, and daily passive range-of-motion (ROM) exercises. Abbreviations: RCT, randomized controlled trial; EVOO, extra virgin olive oil; SICU, surgical intensive care unit; NTICU, neuro-trauma intensive care unit; MICU, medical intensive care unit; NPUAP-EPUAP, National Pressure Ulcer Advisory Panel-European Pressure Ulcer Advisory Panel; AWMA, Australian Wound Management Association

Author (year/country)	Study design	Clinical department/disease	Age, mean (SD)	MV, n (%)	ICU days	Intervention		Control		Measurement
						n	Method	n	Method	
Sönmez and Yapucu Güneş [17] (2020/Turkey)	RCT	Anesthesia and Neurosurgery ICU	Intervention: 61.4 (17.4); Control: 58.5 (19.2)	Intervention: 14 (25.1); Control: 20 (31.2)	5 days ≤	65	EVOO was applied to heels (0.5 mL) twice daily for 5 days to 4 weeks, or until a pressure injury developed, ICU stay	64	No moisturizing products were used	NPUAP-EPUAP
Borzou et al. [18] (2020/Iran)	RCT	Trauma patients, mainly undergoing major surgery	Intervention: 52.7 (19.5); Control: 53.1 (20.0)	NA	NA	72	Sweet almond oil or paraffin was applied to pressure-prone heels (1 mL per heel) daily for 7 days	36	Standard care	NPUAP-EPUAP
Hahnel et al. [19] (2020/Germany)	RCT	ICU in seven departments, including surgery and cardiology	Intervention: 63.8 (15.6); Control: 63.1 (15.2)	Intervention: 199 (93.9); Control: 200 (95.2)	NA	212	Mepilex Border Heel dressings were applied to the heels and renewed every 3 days	210	Standard care	NPUAP-EPUAP
Meyers [9] (2017/the United States)	RCT	SICU, NTICU, MICU	Intervention: 44.6 (17.1); Control: 40.7 (14.9)	NA	NA	37	The heel protectors	17	Standard care	Via a non-validated heel skin assessment tool
Santamaria et al. [20] (2015/Australia)	RCT	Trauma and critically ill patients	Intervention: 54 (20.8); Control: 56 (20.5)	Intervention: 155; Control: 153	Mean (SD) Intervention: 91 (112); Control: 86 (101)	161	Mepilex® Heel dressings were applied to heels with Tubifast® bandages and renewed every 3 days	152	Standard care	AWMA
Santamaria et al. [21] (2015/Australia)	Prospective cohort study	All major trauma and critically ill patients	Intervention: 55 (19.7); Control: 56 (20.5)	Intervention: 127 (23); Control: 153 (29)	Mean (SD) Intervention: 107 (123); Control: 86 (101)	150	Foam heel dressings were applied to both heels and renewed every 3 days	152	Standard care	AWMA
Arslan and Ates [22] (2024/Turkey)	RCT	General and Adult ICUs	Intervention: 72.4 (13.9); Control: 74.5 (13.3)	NA	5 days ≤	42	Heel protector boots	42	Standard care	NPUAP-EPUAP

In all articles, the control group had regular positional changes, retention, rehabilitation, and choice of bed mats as part of their usual care. In the intervention group, in addition to usual care, two of seven studies [17,18] used oil application, three studies [19,20,21] used heel dressing, and two studies [9,22] used protector intervention.

Risk of Bias in the Included Studies

Both RCTs and non-RCTs were assessed using the MMAT (Table 2). For the two common screening questions, all included studies presented “Yes,” and there was a low risk of bias. All six RCTs were adequately randomized, had a comparable baseline for the intervention and control groups, and had complete outcome data. In all studies, the participants could comply with the interventions. One (16.7%) study had outcome assessors blinded to the details of the intervention, three (50.0%) were unblinded, and two (33.3%) did not clearly describe blinding. In one non-RCT study, the participants were well chosen, the variables were measured reliably, and the outcome data were analyzed appropriately. The intervention was implemented throughout the study period. Confounding factors were also considered in the design and analyses. All studies had a bias score of 0%-20%, which indicated a low risk. We also examined publication bias using funnel plots. However, due to the small number of papers included in this study (no more than 10), the shape of the plots was unstable.

**Table 2:** Bias risk assessment of the included studies The methodological quality criteria for the RCTs were as follows: (2.1) Are the study participants appropriately randomized? (2.2) Are the intervention and control groups comparable at the beginning of the study? (2.3) Is complete data available? (2.4) Is the outcome assessor blinded to the intervention? (2.5) Did the participants follow the assigned interventions? (3.1) Is the participant representative of the target population? (3.2) Are the variables measured in a clear and credible manner? (3.3) Is complete result data available? (3.4) Are confounding factors considered in the design and analysis? (3.5) Was the intervention implemented during the study period as intended? Abbreviation: Y, Yes; N, No; C, Can’t tell

Randomized controlled trials		Methodological quality criteria					Risk score (%)
		2.1	2.2	2.3	2.4	2.5	
Santamaria et al. [20]	2015	Y	Y	Y	C	Y	20
Meyers [9]	2017	Y	Y	Y	N	Y	20
Hahnel et al. [19]	2020	Y	Y	Y	Y	Y	0
Sönmez and Yapucu Güneş [17]	2020	Y	Y	Y	N	Y	20
Borzou et al. [18]	2020	Y	Y	Y	C	Y	20
Arslan and Ates [22]	2024	Y	Y	Y	C	Y	20
Non-randomized controlled trials		3.1	3.2	3.3	3.4	3.5	
Santamaria et al. [21]	2015	Y	Y	Y	Y	Y	0

Outcomes

**Figure 2:**
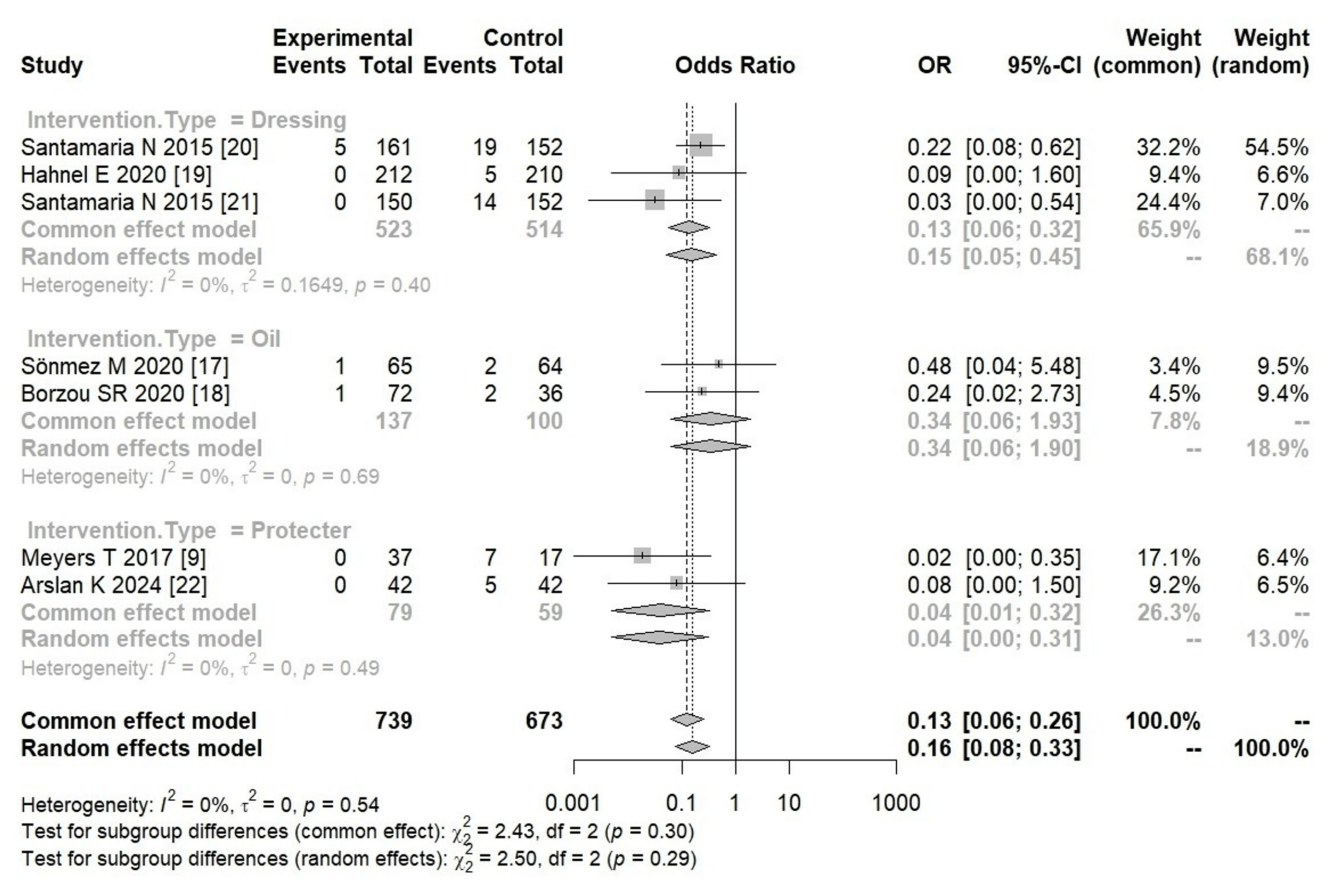
Forest plot of the included studies Forest plot of subgroup analysis based on intervention for the prevention of heel ulcers. Abbreviations: OR, odds ratio; CI, confidence interval; χ2, chi-squared test; df, degree of freedom

The results of the meta-analysis are shown in Figure 2. In this study, additional pressure ulcer preventive interventions on the heel prevented pressure ulcers compared with usual care alone (OR = 0.16, 95% CI = 0.08-0.33, I² = 0%, large effect size). In addition, heterogeneity was low. Analyses were performed on subgroups for each intervention. There were two RCTs [19,20] and one non-RCT [21] that used dressings. The application of the dressing was effective in preventing pressure ulcers compared with normal care alone (OR = 0.15, 95% CI = 0.05-0.45, I² = 0%, large effect size). In addition, there were two studies in which protectors were worn (OR = 0.04, 95% CI = 0.00-0.31, I² = 0%, very large effect size) [9,22]. However, oil application did not show any effect on preventing pressure ulcers compared with usual care alone (OR = 0.34, 95% CI = 0.06-1.90, I² = 0%) [17,18]. All the intervention methods resulted in low heterogeneity.

A sensitivity analysis was performed by study design. Six of the seven studies were RCTs (OR = 0.18, 95% CI = 0.08-0.39, I² = 0%), and one was a non-RCT (OR = 0.03, 95% CI = 0.00-0.54). In the analysis based on design, the results showed that the intervention prevented pressure ulcers. The six RCTs demonstrated low heterogeneity (Appendix Figure 3).

Discussion

Summary of Findings

A systematic review and meta-analysis were conducted to determine the effectiveness of preventive interventions for heel ulcers, and the results were pooled from seven papers, with a total of 1,412 participants. Heel pressure ulcer prevention interventions included three studies using dressings, two using oil applications, and two using protector interventions, and the results showed that these interventions were effective in preventing heel pressure ulcers compared to usual care alone.

Heel pressure ulcers can be easily prevented by simple care, such as wearing a protector and applying a dressing to prevent heel ulcers in severely ill patients. A 2021 meta-analysis of previous studies in patients in acute care hospitals also showed a significant protective effect from wearing protectors, which was consistent (relative risk (RR) = 0.38, p < 0.001) [8]. A meta-analysis of an intervention in which a prophylactic dressing was applied to the heel of critically ill patients found similar results (RR = 0.31, 95% CI = 0.12-0.80, p = 0.02) [7]. On the other hand, in this study, the effect of applying oil to prevent pressure ulcers on the heel was compared for the first time, but the preventive effect of oil was not shown.

Existing Evidence and Gaps

In this study, a subgroup analysis was used to compare protectors and dressings with placebo or normal care alone and found a protective effect. On the other hand, there have been no comparative studies between the types of dressings and protectors, and no studies have been conducted on which types of prevention methods are most effective. Therefore, there is a lack of further comparative studies between interventions that have been found to have such protective effects on the heels of critically ill patients. In addition, pressure ulcer prevention, including for the heels, reduced the mortality of patients with and without pressure ulcers (9.1% vs. 1.8%, OR = 5.08, 95% CI = 5.03-5.1, p < 0.001) and length of hospital stay (median: seven days vs. three days), but also improved patient outcomes, such as reducing healthcare costs (median: $36,500 vs. $17,200) [23]. A comparative study in Brazil in critically ill patients [24] showed the same results for mortality (48.8% vs. 21.6%, p < 0.001) and length of hospital stay (20.7 ± 15.76 days vs. 9.7 ± 8.07 days, p < 0.001). In addition, compared to the care of pressure ulcer treatment, preventive care can be done with less time and effort and prevents the increase in medical burden and medical costs. However, cost-effectiveness was not included in the evaluation of this study. In a previous study, Lyman [25] found that protector wear is effective in preventing heel pressure ulcers and can significantly offset the cost of treatment, although initial costs are required. Also, according to a study by El Genedy et al. [26], the application of prophylactic dressings in critically ill patients is limited in the heel area due to cost-effectiveness, but dressings should be applied for patients at high risk for pressure ulcers. Both studies indicate that the more severe the pressure ulcer, the more cost-effective it is. Therefore, it is necessary to prevent heel pressure ulcers according to the economic situation and culture of the area. From the above, it follows that in the future, it will be necessary to conduct comparative studies between each intervention method from a broad perspective, including not only preventive effects but also patient outcomes and medical burdens.

There were also different types of interventions in the prevention of oils, protectors, and dressings. In the included studies, all three dressings were silicone dressings, with some having three layers and some having five layers. The oils applied in the intervention included extra virgin olive oil, almond oil, and paraffin oil. A comparative study conducted in Iran in 2019 [27] on the prevention of pressure ulcers by type of oil examined the effects of henna oil and olive oil as treatments for pressure ulcer grade I, but it was not clearly shown whether they were effective in preventing pressure ulcers. There are also other comparative studies in which olive oil and aloe vera gel were applied alone or in combination to critically ill patients, showing efficacy in preventing pressure ulcers (OR = 0.43 in the olive group, 0.86 in the aloe vera group, 0.34 in both groups) [28], but it was not an intervention for the heel. In this way, although there are various studies, which type of oil is better has not been compared, and such comparative studies will be necessary in the future.

Implications for Clinical Practice and Research

In this study, it was clarified that, in addition to usual care for the heel, the intervention of applying a protector or a dressing can help prevent pressure ulcers on the heel. This means that the intervention is suitable for patients with unstable circulatory dynamics or those who are immobile due to sedation or intubation management, and that it is possible to prevent heel ulcers by adding simple interventions to existing care. Research suggests that protective patches and dressings are effective in preventing heel pressure ulcers in critically ill patients, but it is not yet known which interventions are most effective. In addition, there are differences in the types of interventions and the materials used (e.g., the type of protector or dressing), and it remains unclear which option is the simplest and most effective. Therefore, such comparative studies need to be conducted through large-scale RCTs.

Strengths and limitations of research

To the best of our knowledge, this is the first report of any systematic review or meta-analysis of pressure ulcer prevention interventions limited to the heel of critically ill patients. Existing meta-analyses have focused on older adults or the acute phase in general, not specifically on higher-risk ICU patients. In this study, we conducted a meta-analysis that included not only RCTs but also non-RCTs. These are the strengths of this study. However, there are also limitations in some of the included studies. First, the inclusion of non-RCTs may lead to bias. In this study, the MMAT assessment showed low risk in all literature, so the risk of bias is judged to be small, and its effect may be minimal. Second, we analyzed the intervention methods as a subgroup analysis, classifying them into dressings, protectors, and oil applications. However, the number of studies was small - two on oil applications, two on protectors, and three on dressings. Due to the small number of studies, caution should be exercised when interpreting the results of the subgroup analyses, and the number of high-quality intervention studies will need to be increased in the future. Third, we were unable to assess publication bias using a funnel plot. This was due to the small number of included papers, which made it difficult to accurately determine the presence or absence of asymmetry or bias. In addition, the results are easily influenced by specific conditions and circumstances, making it difficult to draw broad conclusions. Therefore, the generalizability of the present results is considered limited.

## Conclusions

This meta-analysis demonstrated that additional preventive interventions for heel pressure ulcers in critically ill patients were more effective in preventing the incidence of heel pressure ulcers compared to usual care. The use of dressings and protectors has a preventive effect on heel pressure ulcers. Oil application, however, does not have a preventive effect on pressure ulcers. Additionally, the number of studies is small and insufficient to draw firm conclusions from the subgroup analysis.

## Figures and Tables

**Figure 3:**
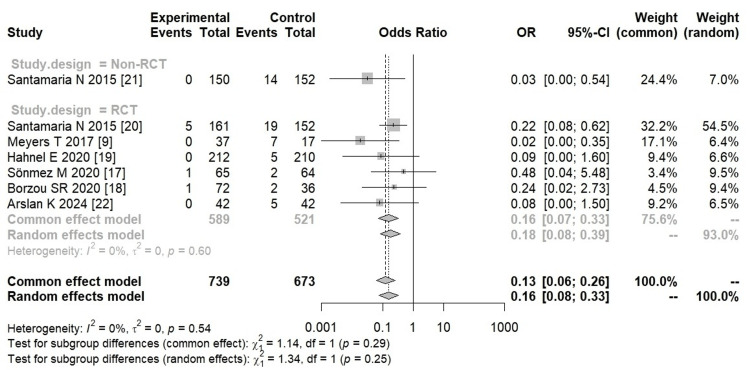
Forest plot by study design A sensitivity analysis based on study design. Abbreviations: OR, odds ratio; CI, confidence interval; χ^2^, chi-squared test; df, degree of freedom

## Appendices

**Table 3:** Preferred Reporting Items for Systematic Reviews and Meta-Analyses 2020 checklist Preferred Reporting Items for Systematic Reviews and Meta-Analyses (PRISMA) were judged according to the 2020 PRISMA checklist.

Section and topic	Item #	Checklist item	Location where item is reported
TITLE			
Title	1	Identify the report as a systematic review.	Page 1
ABSTRACT			
Abstract	2	See the PRISMA 2020 for Abstracts checklist.	-
INTRODUCTION			
Rationale	3	Describe the rationale for the review in the context of existing knowledge.	Pages 3-4
Objectives	4	Provide an explicit statement of the objective(s) or question(s) the review addresses.	Page 4
METHODS			
Eligibility criteria	5	Specify the inclusion and exclusion criteria for the review and how studies were grouped for the syntheses.	Page 5
Information sources	6	Specify all databases, registers, websites, organizations, reference lists, and other sources searched or consulted to identify studies. Specify the date when each source was last searched or consulted.	Page 4
Search strategy	7	Present the full search strategies for all databases, registers and websites, including any filters and limits used.	Page 4
Selection process	8	Specify the methods used to decide whether a study met the inclusion criteria of the review, including how many reviewers screened each record and each report retrieved, whether they worked independently, and if applicable, details of automation tools used in the process.	Page 5
Data collection process	9	Specify the methods used to collect data from reports, including how many reviewers collected data from each report, whether they worked independently, any processes for obtaining or confirming data from study investigators, and if applicable, details of automation tools used in the process.	Page 5
Data items	10a	List and define all outcomes for which data were sought. Specify whether all results that were compatible with each outcome domain in each study were sought (e.g. for all measures, time points, analyses), and if not, the methods used to decide which results to collect.	Page 5
	10b	List and define all other variables for which data were sought (e.g., participant and intervention characteristics, funding sources). Describe any assumptions made about any missing or unclear information.	Page 6
Study risk of bias assessment	11	Specify the methods used to assess risk of bias in the included studies, including details of the tool(s) used, how many reviewers assessed each study and whether they worked independently, and if applicable, details of automation tools used in the process.	Page 6
Effect measures	12	Specify for each outcome the effect measure(s) (e.g., risk ratio, mean difference) used in the synthesis or presentation of results.	Page 5
Synthesis methods	13a	Describe the processes used to decide which studies were eligible for each synthesis (e.g. tabulating the study intervention characteristics and comparing against the planned groups for each synthesis (item #5)).	Page 6
	13b	Describe any methods required to prepare the data for presentation or synthesis, such as handling of missing summary statistics, or data conversions.	Page 6
	13c	Describe any methods used to tabulate or visually display results of individual studies and syntheses.	Page 6
	13d	Describe any methods used to synthesize results and provide a rationale for the choice(s). If meta-analysis was performed, describe the model(s), method(s) to identify the presence and extent of statistical heterogeneity, and software package(s) used.	Page 6
	13e	Describe any methods used to explore possible causes of heterogeneity among study results (e.g. subgroup analysis, meta-regression).	Page 6
	13f	Describe any sensitivity analyses conducted to assess robustness of the synthesized results.	Page 6
Reporting bias assessment	14	Describe any methods used to assess risk of bias due to missing results in a synthesis (arising from reporting biases).	Page 6
Certainty assessment	15	Describe any methods used to assess certainty (or confidence) in the body of evidence for an outcome.	Page 6
RESULTS			
Study selection	16a	Describe the results of the search and selection process, from the number of records identified in the search to the number of studies included in the review, ideally using a flow diagram.	Page 6
	16b	Cite studies that might appear to meet the inclusion criteria, but which were excluded, and explain why they were excluded.	Page 7
Study characteristics	17	Cite each included study and present its characteristics.	Page 7
Risk of bias in studies	18	Present assessments of risk of bias for each included study.	Page 7
Results of individual studies	19	For all outcomes, present, for each study: (a) summary statistics for each group (where appropriate) and (b) an effect estimate and its precision (e.g. confidence/credible interval), ideally using structured tables or plots.	Page 8
Results of syntheses	20a	For each synthesis, briefly summarize the characteristics and risk of bias among contributing studies.	Page 8
	20b	Present results of all statistical syntheses conducted. If meta-analysis was done, present for each the summary estimate and its precision (e.g. confidence/credible interval) and measures of statistical heterogeneity. If comparing groups, describe the direction of the effect.	Page 8
	20c	Present results of all investigations of possible causes of heterogeneity among study results.	Page 8
	20d	Present results of all sensitivity analyses conducted to assess the robustness of the synthesized results.	Page 8
Reporting biases	21	Present assessments of risk of bias due to missing results (arising from reporting biases) for each synthesis assessed.	Page 8
Certainty of evidence	22	Present assessments of certainty (or confidence) in the body of evidence for each outcome assessed.	Page 8
DISCUSSION			
Discussion	23a	Provide a general interpretation of the results in the context of other evidence.	Pages 8-9
	23b	Discuss any limitations of the evidence included in the review.	Page 10
	23c	Discuss any limitations of the review processes used.	Page 10
	23d	Discuss implications of the results for practice, policy, and future research.	Page 9
OTHER INFORMATION			
Registration and protocol	24a	Provide registration information for the review, including the register name and registration number, or state that the review was not registered.	Page 4
	24b	Indicate where the review protocol can be accessed, or state that a protocol was not prepared.	-
	24c	Describe and explain any amendments to information provided at registration or in the protocol.	-
Support	25	Describe sources of financial or non-financial support for the review, and the role of the funders or sponsors in the review.	Page 13
Competing interests	26	Declare any competing interests of review authors.	Page 13
Availability of data, code and other materials	27	Report which of the following are publicly available and where they can be found: template data collection forms; data extracted from included studies; data used for all analyses; analytic code; any other materials used in the review.	Page 13

**Table 4:** Search terms This table indicates the search formula used for the literature search in accordance with the search criteria.

Database	Search terms
PubMed	Intensive care units[mh] OR critical care[mh] OR critical illness[mh] OR "ICU"[tiab] OR intensive care unit*[tiab] OR "intensive care"[tiab] OR "critical care"[tiab] OR "critical illness"[tiab] OR "critically ill"[tiab] AND pressure ulcer [mh] OR leg ulcer [mh] OR foot ulcer [mh] OR foot diseases [mh] OR "pressure ulcer*" [tiab] OR "pressure sore*" [tiab] OR "pressure injur*" [tiab] OR "Decubitus Sore*" [tiab] OR "Decubitus Ulcer*" [tiab] OR "bed sore*"[tiab] OR "leg ulcer*" [tiab] OR "foot ulcer*"[tiab] OR "deep tissue injur*"[tiab] OR "deep tissue ulcer*"[tiab] OR "heel ulcer*"[tiab] OR "heel pain*"[tiab] OR "foot disease*"[tiab] AND "Random Allocation"[mh] OR "Controlled Clinical Trials as Topic"[mh] OR "Controlled Before-After Studies"[mh] OR "non-Randomized Controlled Trials as Topic"[mh] OR "clinical Trials as Topic"[mh] OR "placebos"[mh] OR "Drug Therapy"[mh] OR "Control Groups"[mh] OR "Quality improvement"[mh] OR "Randomized Controlled Trial"[pt] OR "controlled clinical trial"[pt] OR "drug therapy"[sh] OR "Random Allocation*"[tiab] OR "Random*"[tiab] OR "Random Assignment*"[tiab] OR "randomized controlled trial*"[tiab] OR "controlled clinical trial"[tiab] OR "RCT"[tiab] OR "trial*"[tiab] OR "Controlled Before-After Stud*"[tiab] OR "Before-After trial*" [tiab] OR "pre-post comparative stud*"[tiab] OR "non-Randomized Controlled Trial*"[tiab] OR "NRCT"[tiab] OR "Experimental Stud*"[tiab] OR "intervention stud*"[tiab] OR "placebo*"[tiab] OR "Sham Treatment*"[tiab] OR "Chemotherap*"[tiab] OR "Pharmacotherap*"[tiab] OR "Control Group*"[tiab] OR "Quality improvement"[tiab] OR "improvement project"[tiab] NOT animls [mh] NOT humans [mh]
Cochrane Central	(MeSH descriptor:[intensive care unit]explode all trees) OR (MeSH descriptor:[critical care]explode all trees) OR (MeSH descriptor:[critical illness]explode all trees) OR (ICU:ti,ab,kw) OR ("intensive care unit*":ti,ab,kw) OR ("intensive care":ti,ab,kw) OR ("critical care ":ti,ab,kw) OR ("critical illness ":ti,ab,kw) OR ("critically ill":ti,ab,kw) AND (MeSH descriptor:[pressure ulcer]explode all trees) OR (MeSH descriptor:[leg ulcer]explode all trees) OR (MeSH descriptor:[foot ulcer]explode all trees) OR (MeSH descriptor:[foot diseases]explode all trees) OR ("pressure ulcer*":ti,ab,kw) OR ("pressure sore*":ti,ab,kw) OR ("pressure injur*":ti,ab,kw) OR ("Decubitus Sore*":ti,ab,kw) OR ("Decubitus Ulcer*":ti,ab,kw) OR ("bed sore*":ti,ab,kw) OR ("bed ulcer*":ti,ab,kw) OR ("leg ulcer*":ti,ab,kw) OR ("foot ulcer*":ti,ab,kw) OR ("deep tissue injur*":ti,ab,kw) OR ("deep tissue ulcer*":ti,ab,kw) OR ("deep tissue sore*":ti,ab,kw) OR ("heel ulcer*":ti,ab,kw) OR ("heel sore*":ti,ab,kw) OR ("heel pain*":ti,ab,kw) OR ("foot disease*":ti,ab,kw) AND (MeSH descriptor:[Random Allocation]explode all trees) OR (MeSH descriptor:[randomized controlled trial]explode all trees) OR (MeSH descriptor:[Randomized Controlled Trials as Topic]explode all trees) OR (MeSH descriptor:[Controlled Clinical Trial]explode all trees) OR (MeSH descriptor:[Controlled Clinical Trials as Topic]explode all trees) OR (MeSH descriptor:[Controlled Before-After Studies]explode all trees) OR (MeSH descriptor:[Clinical Trials as Topic]explode all trees) OR (MeSH descriptor:[placebos]explode all trees) OR (MeSH descriptor:[Drug Therapy]explode all trees) OR (MeSH descriptor:[Control Groups]explode all trees) OR (MeSH descriptor:[Quality improvement]explode all trees) OR ("Random Allocation":ti,ab,kw) OR ("Random*":ti,ab,kw) OR ("Random Assignment*":ti,ab,kw) OR ("randomized controlled trial*":ti,ab,kw) OR ("RCT":ti,ab,kw) OR ("Controlled Before-After Stud*":ti,ab,kw) OR ("Before-After trial*":ti,ab,kw) OR ("pre-post comparative stud*":ti,ab,kw) OR ("non-Randomized Controlled Trial*":ti,ab,kw) OR ("NRCT":ti,ab,kw) OR ("trial*":ti,ab,kw) OR ("Experimental Stud*":ti,ab,kw) OR ("intervention Stud*":ti,ab,kw) OR ("placebo*":ti,ab,kw) OR ("Sham Treatment*":ti,ab,kw) OR ("drug therapy*":ti,ab,kw) OR ("Chemotherap*":ti,ab,kw) OR ("Pharmacotherap*":ti,ab,kw) OR ("Control Group*":ti,ab,kw) OR ("Quality improvement":ti,ab,kw) OR ("improvement project":ti,ab,kw) NOT ("animals ":ti,ab,kw) NOT ("humans":ti,ab,kw)
CINAHL	(MH Intensive care units) OR (MH critical care) OR (MH critical illness) OR ("ICU") OR ("intensive care unit*") OR ("intensive care") OR ("critical care") OR ("critical illness") OR ("critically ill") AND (MH"pressure ulcer") OR (MH"leg ulcer") OR (MH"foot ulcer") OR (MH"deep tissue injury") OR (MH "heel ulcer") OR (MH"foot Diseases") OR ("pressure ulcer*") OR ("pressure sore*") OR ("pressure injur*") OR ("Decubitus ulcer") OR ("bed sore*") OR ("leg ulcer*") OR ("foot ulcer*") OR ("deep tissue injur*") OR ("heel ulcer") OR ("heel sore*") OR ("heel pain*") OR ("foot disease*") AND (MH“Random Assignment") OR (MH"randomized controlled trials") OR (MH"Controlled Before-After Studies") OR (MH"clinical trials") OR (MH"Experimental Studies"(MH"placebos") OR (MH"drug therapy") OR (MH"Control Group") OR (MH"Quality improvement") OR ("Random Assignment*") OR ("Random Allocation*") OR ("Random*") OR ("randomized controlled trial*") OR ("RCT") OR ("Controlled Before-after stud*") OR ("Before-After trial*") OR ("non-Randomized Controlled Trial*") OR ("trial*") OR ("Experimental Stud*") OR ("intervention Stud*") OR ("placebo*") OR ("Sham Treatment*") OR ("drug therapy*") OR ("Chemotherap*") OR ("Pharmacotherap*") OR ("Control Group*") OR ("improvement project") NOT (MH animals) NOT (MH humans)
Igaku Chuo Zasshi	ICU/TH OR クリティカルケア/TH OR 危篤/TH OR ICU/TA OR 集中治療室/TA OR 集中治療/TA OR クリティカルケア/TA OR 危篤/TA OR 重症/TA OR 重病/TA OR 重症疾患/TA AND 褥瘡性潰瘍/TH OR 下肢潰瘍/TH OR 足部潰瘍/TH OR 足部疾患/TH OR 褥瘡性潰瘍/TA OR 褥瘡/TA OR 圧迫性潰瘍/TA OR 褥創/TA OR 床ずれ/TA OR とこずれ/TA OR 下肢潰瘍/TA OR 足部潰瘍/TA OR 足潰瘍/TA OR 足の潰瘍/TA OR 深部組織/TA OR 踵潰瘍/TA OR 踵の痛み/TA OR 足部疾患/TA OR 足病変/TA AND ランダム割付け/TH OR ランダム化比較試験/TH OR 臨床試験/TH OR 介入研究/TH OR プラセボ/TH OR 薬物療法/TH OR 対照群/TH OR ランダム/TA OR 確率化/TA OR 無作為化/TA OR RCT/TA OR 前後比較研究/TA OR 前後比較試験/TA OR 前後研究/TA OR 前後試験/TA OR 臨床試験/TA OR 非盲検/TA OR 介入研究/TA OR プラセボ/TA OR 偽薬/TA OR 投薬/TA OR 化学療法/TA OR 薬物治療/TA OR 与薬/TA OR 対照/TA OR 医療の質改善/TA NOT 動物/TH NOT ヒト/TH
